# Supplementary material for: FERN – a Java framework for stochastic simulation and evaluation of reaction networks
Source: BMC Bioinformatics. 2008 Aug 29;9:356. doi: 10.1186/1471-2105-9-356 (PMC2553347; doi:10.1186/1471-2105-9-356)
Supplement: Additional file 1 — FERN distribution, Version 1.3. This archive contains the FERN source code and binaries as well as documentation and example models in FernML and SBML. [file 1471-2105-9-356-S1.zip › fern/doc/javadoc/fern/tools/functions/Probability.Constant.html]

Probability.Constant


---


|  |  |  |
| --- | --- | --- |
| **Overview** \| **Package** \| **Class** \| **Use** \| **Tree** \| **Deprecated** \| **Index** \| **Help** | |  |
| **PREV CLASS**   **NEXT CLASS** | **FRAMES**    **NO FRAMES**     **All Classes** |  |
| SUMMARY: NESTED \| FIELD \| CONSTR \| METHOD | DETAIL: FIELD \| CONSTR \| METHOD |  |


---


## fern.tools.functions Class Probability.Constant

```
java.lang.Object
  fern.tools.functions.Probability.Constant
```

**All Implemented Interfaces:**: Probability

**Enclosing interface:**: Probability

---

``` public static class Probability.Constant extends Object implements Probability ```

Implementation that yields a constant (and hence independent)
probability.

**Author:**
:   Florian Erhard

---

**Nested Class Summary**

**Nested classes/interfaces inherited from interface fern.tools.functions.Probability**

`Probability.Constant, Probability.ReactionProbability`


**Constructor Summary**

`Probability.Constant(double p)`


| **Method Summary** | |
| --- | --- |
| `double` | `getProb(int l1, int l2)`             Gets the probability depending on two integers. |

**Methods inherited from class java.lang.Object**

`clone, equals, finalize, getClass, hashCode, notify, notifyAll, toString, wait, wait, wait`

**Constructor Detail**

### Probability.Constant

```
public Probability.Constant(double p)
```


**Method Detail**

### getProb

```
public double getProb(int l1,
                      int l2)
```

:   **Description copied from interface: `Probability`**
:   Gets the probability depending on two integers.

    :   **Specified by:**: `getProb` in interface `Probability`
    :   **Parameters:**: `l1` - interger 1: `l2` - integer 2 **Returns:**: probability


---


|  |  |  |
| --- | --- | --- |
| **Overview** \| **Package** \| **Class** \| **Use** \| **Tree** \| **Deprecated** \| **Index** \| **Help** | |  |
| **PREV CLASS**   **NEXT CLASS** | **FRAMES**    **NO FRAMES**     **All Classes** |  |
| SUMMARY: NESTED \| FIELD \| CONSTR \| METHOD | DETAIL: FIELD \| CONSTR \| METHOD |  |


---
